# That lung cancer incidence falls in ex-smokers: misconceptions 2

**DOI:** 10.1038/sj.bjc.6606080

**Published:** 2011-02-01

**Authors:** J Peto

**Affiliations:** 1 London School of Hygiene and Tropical Medicine, Keppel St., London WC1E 7HT, UK

## Abstract

Misconceptions and ill-founded theories can arise in all areas of science. However, the apparent accessibility of many epidemiology findings and popular interest in the subject can lead to additional misunderstandings. The article below continues an occasional series of short editorials highlighting some current misinterpretations of epidemiological findings. Invited authors will be given wide scope in judging the prevalence of the misconception under discussion. We hope that this series will prove instructive to cancer researchers in other disciplines as well as to students of epidemiology. *Adrian L Harris* and *Leo Kinlen*

There is a widespread misconception in the general population, and even among some epidemiologists, that the incidence rate of lung cancer declines in ex-smokers. In fact, when smoking ceases, the rate stops increasing steeply and remains almost constant (Figure 1: Halpern *et al*, 1993). This misconception presumably arose because the *relative* risk falls rapidly in ex-smokers, as it is calculated by dividing the roughly constant ex-smokers’ rate by the rising rate in non-smokers. (Whether the slight increase in incidence after stopping smoking is greater than the increase in non-smokers of the same age, as Figure 1 suggests, may never be known. Many ex-smokers relapse, and some may fail to admit it.) The lifelong increased risk in those who started smoking when they were very young indicates that smoking initiates lung carcinogenesis, but the incidence pattern in ex-smokers is particularly informative. The immediate effect of stopping suggests that smoking also acts at a late stage in carcinogenesis, but as the rate does not fall when smoking ceases it seems that the final event that a cell must undergo to become fully malignant is unaffected by smoking (Cairns, 2006). The age distribution of cancer, and particularly of lung cancer in smokers and non-smokers, led to multi-stage models of carcinogenesis long before altered genes were observed in human cancer (Armitage and Doll, 1954; Doll, 1978). Various alternative models have been proposed (Altshuler, 1989; Samet *et al*, 2007), and which (if any) is correct must ultimately be decided from molecular rather than statistical studies. Molecular biologists should, however, be aware of these epidemiological observations, as they must be relevant to understanding the significance of the somatic changes in lung cancer that are now being discovered (Pleasance *et al*, 2010).

## Figures and Tables

**Figure 1:**
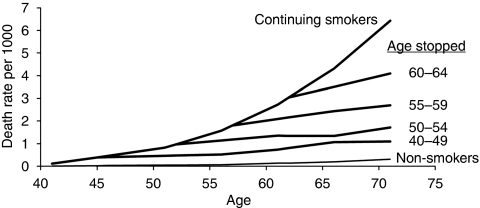
Lung cancer mortality in continuing smokers, ex-smokers and non-smokers. Data from Halpern *et al* (1993).
